# Evaluation of acupuncture for the treatment of pain associated with naturally-occurring osteoarthritis in dogs: a prospective, randomized, placebo-controlled, blinded clinical trial

**DOI:** 10.1186/s12917-020-02567-1

**Published:** 2020-09-25

**Authors:** Alice Baker-Meuten, Theresa Wendland, Shelly K. Shamir, Ann M. Hess, Felix Michael Duerr

**Affiliations:** 1 Colorado State University Veterinary Teaching Hospital, 300 W Drake Rd, Fort Collins, CO 80523 USA; 2 University of Minnesota Veterinary Medical Center, 1365 Gortner Ave, St Paul, MN 55108 USA; 3 Department of Statistics, Colorado State University, 300 W Drake Rd, Fort Collins, CO 80523 USA

**Keywords:** Osteoarthritis, Acupuncture, Canine, Electro-acupuncture, Accelerometers, Objective gait analysis

## Abstract

**Background:**

Acupuncture has been used as a treatment for pain associated with osteoarthritis (OA) for thousands of years; however, there is a lack of definitive evidence for this indication in humans or animals. The aim of this study was to prospectively evaluate the efficacy of acupuncture on lameness and clinical function in dogs affected by naturally-occurring OA using objective outcome measures. A total of 32 client-owned dogs completed this prospective, randomized, placebo-controlled, blinded clinical trial, using a cross-over design. Participants were assigned to receive placebo or acupuncture treatment once weekly for 4 weeks in random order with a two-week wash-out period in between treatment phases. Outcome measures included ground reaction forces (GRF), subjective orthopedic scoring (SOS), activity counts (AC), and owner-completed clinical metrology instruments (CMI; Canine Brief Pain Inventory [CBPI] and Client Specific Outcome Measures [CSOM]). For statistical comparison, baseline GRF, SOS, and CMI data were compared to data obtained 1 week after each treatment phase. Similarly, total weekly AC of the final week of each treatment phase were compared to the baseline week.

**Results:**

Evidence of differences between baseline versus acupuncture and placebo treatments was not identified for the following outcome measures: GRF, AC, or SOS. However, evidence of differences was identified for some of the CMI scores, including the CSOM questionnaire which showed evidence of improvement when comparing baseline versus acupuncture (*p* = 0.0002) as well as between placebo versus acupuncture treatments (*p* = 0.035) but not between baseline versus placebo treatments (*p* = 0.221).

**Conclusions:**

The applied acupuncture protocol did not show improvement in function when using objective outcome measures for OA in dogs; however, certain CMI measurements recorded some degree of treatment response.

## Background

Osteoarthritis (OA) affects humans and animals and is the most common joint disorder in the world [1]. Recent primary care data suggests that clinical OA is observed in approximately 5% of the canine population [2–4]. Furthermore, in an epidemiologic study investigating more than 12,000 German Shepherd Dogs in the UK, OA/musculoskeletal disease was the most common cause of euthanasia or natural death, surpassing even neoplasia [2].

Standard treatments for canine OA include non-steroidal anti-inflammatory drugs (NSAIDs), other oral pain medications, and nutraceuticals [5]. Non-steroidal anti-inflammatory drugs are considered the most effective oral pain medication available and are therefore widely used [6]. Unfortunately, some patients suffer from diseases that preclude the use of traditional NSAIDs and newer NSAIDs such as piprant drugs [7]. Furthermore, traditional NSAIDs have been associated with renal, gastrointestinal, and hepatic toxicity. Due to the fear of these side effects, some owners and veterinarians elect to pursue alternative or additional methods for treating OA, such as acupuncture [6–9].

Acupuncture originated as a part of traditional Chinese medicine and involves stimulation of specific points in the body (acupuncture points). There are many different methods of stimulating acupuncture points; however, acupuncture most commonly involves the placement of sterile needles into acupuncture points (i.e. defined by palpable anatomic locations) [8, 10]. Additional stimulation of the inserted needles, including applying an electrical current in case of electro-acupuncture (EA), have been used to increase the effect [8, 10–12]. The mechanism of action for acupuncture is complex, involving local mechanical effects, and the modulation of peripheral and central nervous system pain signaling pathways [8, 10]. For example, acupuncture has been shown to activate afferent nerve fibers (A-beta, A-delta, and C fibers) and regulate signaling molecules such as opioid peptides, glutamate, 5-hydroxytryptamine, and cholecystokinin to mitigate pain [10, 13, 14].

Available research investigating acupuncture for the alleviation of OA-associated pain does not draw firm conclusions regarding its efficacy in either veterinary or human medicine. Several studies have shown evidence of improvement in people receiving acupuncture; however, the benefits appear to be small and below the clinically relevant threshold [15, 16]. Acupuncture research in people is limited by an inability to perform effective blinding, since the treatment involves the physically noticeable aspect of inserting and manipulating the acupuncture needles [17]. Several studies have investigated the effectiveness of acupuncture for the treatment of canine OA [11, 18–22]. Some of these studies have utilized objective outcome measures; however, all authors acknowledged that further research is necessary. There has also been a systematic review in 2006 and a scoping review in 2017 on the efficacy of acupuncture in veterinary medicine, both of which concluded a need for more high-quality, randomized, controlled trials investigating the use of acupuncture in animals [11, 23].

The purpose of this study was to prospectively evaluate the efficacy of acupuncture to mitigate pain and improve function in dogs affected by naturally-occurring OA. The hypothesis was that acupuncture would result in decreased lameness and improved function when compared to placebo treatment.

## Results

Seventy-two dogs were evaluated for enrollment, 36 dogs qualified and were enrolled in the study. There were 15 male castrated, 3 male intact, 17 female spayed, and 1 female intact dogs. The median age was 10 years (1.5–14 years). The range of enrollment weight was 9–47.5 kg (average 30.88 kg, SD ±8.7) and completion weight range was 9–48.4 kg (average 30.96, SD ±8.8), which was not significantly different (*p* = 0.668). There were 9 Labrador Retrievers, 4 Golden Retrievers, 2 German Shepherd Dogs, 1 Chesapeake Bay Retriever, 1 Newfoundland, 1 Siberian Husky, 1 West Highland Terrier, 1 English Springer Spaniel, 1 Entlebucher, and 15 mixed breed dogs. Thirty-two dogs had bilateral or multiple joint OA and 4 dogs had single joint OA with the following distribution of the primarily affected joint: elbow (*n* = 13), hip (*n* = 11), stifle (*n* = 8), tarsal (*n* = 2), and carpal (*n* = 2). See supplementary material for specifics on individual dogs.

Of the 36 patients enrolled in the clinical trial, four patients had incomplete or omitted data. One patient had completely omitted data due to not following the study protocol, one patient was removed from the study before the second phase (treatment) due to the development of inflammatory bowel disease necessitating treatment with immunosuppressive therapy, one patient had omitted data during the second phase (placebo) due to cervical disc disease causing forelimb lameness, and one dog would not tolerate the acupuncture protocol (despite the initial needle test at GV20 prior to enrollment) and was removed from the study during the second phase (treatment). Therefore, 32 dogs had complete data available for final analysis and 4 dogs had incomplete data consisting only of phase 1, which was included in the analysis. Four dogs were sensitive to needling (vocalizing, displaying anxiety about needle placement) but were able to complete their treatments without force or excessive restraint. The owner of one dog reported generalized soreness after his first acupuncture session, but not after subsequent sessions or during the placebo phase. No adverse effects related to the acupuncture were observed or reported in any of the remaining dogs.

Evidence of differences was not identified for comparisons of baseline to the placebo and treatment phases or between placebo and treatment phases for any of the comparisons for the OGA, AC, or subjective orthopedic scores (SOS) data (Table 1). Based on Bonferroni adjusted F-tests (with *p* < 0.05), evidence of differences was identified for several CMI comparisons. Specifically, client specific outcome measure (CSOM) data showed evidence of a difference between baseline and treatment as well as between treatment and placebo (Table 1). There was also a significant difference observed between baseline and treatment for CBPI PSS and CBPI PIS data; however, significant differences were not observed for the comparison between treatment and placebo. For the latter, a statistically significant difference was also observed during the placebo phase and the differences observed here were confirmed with the Bonferroni adjusted F-test.

**Table 1:** Statistical Analysis Results: Comparison of baseline to treatment and placebo, and between treatment and placebo for all outcome measures

	Baseline Mean (± SE)	Treatment	Treatment/Placebo Mean (± SE)	Tukey *p*-value between baseline and treatment	Tukey *p*-value between treatment and placebo	Bonferroni corrected F-test
%BWD	20.5 (± 0.74)	Acupuncture	20.8 (± 0.87)	0.73	0.96	0.58^a^
		Placebo	21.0 (± 0.83)	0.55		
PVF	40.3 (± 2.1)	Acupuncture	40.1 (± 2.7)	0.97	0.99	1
		Placebo	40.6 (± 2.4)	0.94		
VI	6.75 (± 0.41)	Acupuncture	6.53 (± 0.44)	0.09	0.95	1
		Placebo	6.55 (± 0.38)	0.17		
ASI PVF Contralateral	16.4 (± 2.8)	Acupuncture	15.2 (± 2.8)	0.31	0.96	1
		Placebo	14.9 (± 3.0)	0.46		
ASI PVF Ipsilateral	21.2 (± 1.9)	Acupuncture	22.4 (± 1.8)	0.54	0.99	1
		Placebo	21.8 (± 1.6)	0.46		
ASI PVF Diagonal	22.0 (± 2.0)	Acupuncture	23.7 (± 1.8)	0.35	0.94	1
		Placebo	23.3 (± 1.3)	0.19		
ASI VI Contralateral	16.0 (± 2.6)	Acupuncture	15.6 (± 2.9)	0.84	0.97	1
		Placebo	15.6 (± 2.6)	0.94		
ASI VI Ipsilateral	27.2 (± 1.6)	Acupuncture	27.0 (± 2.0)	0.92	0.93	1
		Placebo	26.8 (± 1.7)	0.10		
ASI VI Diagonal	26.9 (± 2.0)	Acupuncture	27.6 (± 2.0)	0.80	0.95	1
		Placebo	27.4 (± 1.9)	0.61		
Total AC^b^	1,065,154 (± 262,894)	Acupuncture	1,057,267 (± 241,479)	0.31	0.84	1
		Placebo	1,137,379 (± 351,793)	0.67		
AC Sedentary	8083 (± 152)	Acupuncture	8051 (± 138)	0.13	0.76	1
		Placebo	8045 (± 165)	0.47		
AC Light	1250 (± 61)	Acupuncture	1246 (± 61)	0.76	0.79	1
		Placebo	1218 (± 70)	0.10		
AC Moderate	813 (± 98)	Acupuncture	760 (± 81)	0.75	0.68	1
		Placebo	775 (± 101)	0.99		
SOS	9.76 (± 0.53)	Acupuncture	9.27 (± 0.50)	0.035*	0.14	0.58
		Placebo	9.76 (± 0.54)	0.80		
CBPI PSS	4.04 (± 0.29)	Acupuncture	3.34 (± 0.32)	0.008*	0.343	0.18
		Placebo	3.60 (± 0.36)	0.213		
CBPI PIS	4.67 (± 0.36)	Acupuncture	3.50 (± 0.36)	0.0001*	0.257	0.003*
		Placebo	3.85 (± 0.42)	0.018*		
CSOM	3.16 (± 0.11)	Acupuncture	2.66 (± 0.11)	0.0002*	0.035*	0.005*
		Placebo	2.93 (± 0.13)	0.221		

*indicates evidence of a difference (based on *p* < 0.05; not considering Bonferoni adjustment)

^a^Bonferoni adjusted *p*-values are shown for all variables except BWD (primary response variable) where the raw unadjusted *p*-value is shown

^b^Total AC was log10 transformed to satisfy model assumptions, but summary statistics are shown on original scale

The questionnaire regarding owner assessment of treatment efficacy did not show evidence of a difference between treatment and placebo for behavior (median difference for acupuncture versus placebo is 0, Bonferroni adjusted Wilcoxon *p* = 0.51) or clinical signs (median difference for acupuncture versus placebo is − 1, Bonferroni adjusted Wilcoxon *p* = 0.15). For the question regarding which phase of the study the owner thought their dog was receiving acupuncture, 58% (*n* = 19) of owners guessed the phase correctly, 21% guessed incorrectly (*n* = 7), 12% did not see a difference (*n* = 4), and 9% were unsure (*n* = 3).

## Discussion

We rejected our hypothesis because we were unable to detect evidence of differences in the objective outcome measures OGA, SOS, and AC. However, some treatment response was found using owner completed CMI to assess clinical function and chronic pain. These results should be cautiously interpreted while considering the natural fluctuations in the disease process of OA as well as the caregiver placebo effect. Given that owners were blinded to the treatment allocation, our results are suggestive of a possible treatment effect of acupuncture in dogs with OA, which is consistent with previous canine studies and human data [15, 16, 18, 19, 22]. Several factors may explain the lack of evidence of differences for objective outcome measures including the true lack of a treatment effect of the utilized acupuncture protocol, variable responses of individuals to acupuncture, limitations of the utilized outcome measures to detect small differences in canines with OA, and the timing of outcome measurements.

Research investigating the effectiveness of acupuncture for treatment of OA in people has shown that the observed treatment benefits are small, therefore necessitating large sample sizes to detect differences. In canine OA research, most studies are designed to detect a difference similar to what is expected when treating patients with NSAIDs [5, 7, 9]. Furthermore, in many of these studies, patients do not receive additional treatments allowing to more clearly evaluate the treatment benefit. It may not be reasonable to expect acupuncture to exert a treatment effect of a similar magnitude to NSAID therapy; however, Teixeira, et al. found that owners perceived an improvement that lasted longer for acupuncture than NSAIDs [22]. In a clinical setting, acupuncture is most frequently employed as an additional strategy combined with many other conventional therapies; therefore, our study was designed to investigate the additive benefits of acupuncture and as such, most of the study participants were already on a regular medical management protocol that remained unchanged prior to and for the duration of the study period, often consisting of several treatments. While this approach is clinically relevant, it likely made it more difficult to detect evidence of differences between treatment groups.

People have been shown to have inherited genetic factors determining the density of cholecystokinin (CCK) receptors in their bodies and both the release of CCK and CCK receptor density are closely associated with an individual’s response to acupuncture [13]. It has also been demonstrated that there are differences in individual descending inhibitory pain controls and differences in processing physiologic pain and pathologic pain, which may contribute to the varying responses seen clinically and in randomized-controlled trials [13]. It has been proposed that this variability in individual responses to acupuncture due to physiologic mechanisms and genetic diversity amongst the population indicates that there are “responders” and “non-responders” to acupuncture analgesia [13]. It is possible that similar genetic diversity exists in dogs. The present study did not control for the possibility of responder versus non-responder patients since this phenomenon has not been objectively identified in dogs at this time.

In people, the interaction between the therapist and patient itself contributes to the observed placebo effects of sham acupuncture [17]. It is unclear how much of a role this interaction plays in dogs, but it can be theorized to be smaller since dogs are considered to be unaware of the treatment purpose. This, in combination with the inability to effectively blind people to acupuncture treatments, makes the canine an ideal translational model to investigate different acupuncture protocols. It is important to note that a substantial caregiver placebo effect has been described in dogs [24], which could explain the evidence of differences identified for CBPI pain interference score (PIS) between baseline and treatment and between baseline and placebo, but not between the treatment and placebo treatments. In other words, since owners were blinded to when their dog was receiving acupuncture (and likely believed in acupuncture since enrolment in the study was voluntary), the positive effect during the placebo treatment is likely due to caregiver placebo effect. It should also be noted that these patients received similar attention from the staff involved in the study during their placebo phase which may have affected their behavior; however, this was intentional to provide each dog with a similar hospital experience with the only variable being the acupuncture and acupuncturist.

Evaluation of at-home physical activity has been suggested as an important outcome measure for chronic diseases such as OA in people and dogs [25, 26]. The Actical accelerometer^b^ was used in this study since it has been previously validated as an outcome measure for physical activity monitoring in dogs [26–28]. An increase in 20% of weekly AC has been observed in dogs receiving NSAID therapy for OA compared with placebo [26]. While it has been described that the total amount of precipitation and daylight hours are unlikely to produce a clinically relevant change in activity data [29], that study did not evaluate other owner-induced factors that have been proposed to change daily AC such as availability to spend time with their dog and motivation to encourage activity [28]. We attempted to address some of the shortcomings of activity monitoring by measuring changes in the amount of sedentary time, light, moderate, and/or vigorous activity times [28]. However, we were unable to detect evidence of change in any of the activity categories in our population during either phase of the study. A recent report on functional linear modeling of activity data in dogs showed improvement in sleep in dogs with OA treated with meloxicam compared to placebo in a cross-over design study [30]. Their conclusions indicate that functional linear modeling is a more sensitive technique for analyzing activity data in dogs than traditional summary techniques. If further research suggests the validity of this technology it could be considered for future studies.

We chose %BWD as our primary outcome measure since it has been suggested to be the most accurate gait parameter when evaluating a heterogeneous group at a controlled velocity, and was associated with a lower variability than peak vertical force (PVF%) [31]. However, regardless of which OGA data point is utilized, ground reaction forces (GRF) only represent a short time point within the dog’s daily status and the data may be influenced by many variables (e.g. variability of OA-associated pain, level of prior activity etc.). Previous studies on acupuncture for OA in dogs also have not shown an improvement in OGA, yet have found differences in CMI scores [19, 22]. To date, there is a lack of objective outcome measures that reliably detect the clinical function of dogs in their natural environment over a longer time period. Until such outcome measures are available, CMIs are the most established method to assess this important feature of OA in canines. Future research is needed that allows for long-term measurement of kinetic parameters in the participant’s natural environment.

We chose to evaluate outcomes 1 week after the last acupuncture treatment. This decision was based on clinical relevance and previous research indicating that the peak benefits of acupuncture are observed after several once weekly treatments [18–22], and lasted up to 2 weeks following the final treatment in one study comparing NSAID therapy to acupuncture for hip OA [22]. Our results support those findings, since owners perceived an improvement 1 week after the final acupuncture treatment. Furthermore, similar to Teixeira et al. [22], we were unable to confirm this benefit with our primary outcome measure (OGA), it is therefore possible that the timing was past the maximum treatment effect. We may have observed greater differences if we would have implemented our time points closer to the last treatment. On the other hand, for a treatment to be clinically relevant for palliation of chronic disease, a shorter treatment effect may not be clinically meaningful.

In traditional Chinese medicine, acupuncture points are tailored to the patient’s pattern diagnosis and not necessarily to the Western diagnosis of OA. In practice, acupuncture protocols following Chinese or Western medicine techniques, are very dynamic and are frequently changed based on subjective assessment of how the patient responded to their previous treatment. This dynamism was confirmed in our survey. In our attempt to minimize confounding factors, we chose a set protocol based on treating the most severely or clinically affected joint. However, this approach does not necessarily represent the clinical treatment performed in daily practice. Similarly, some patients do not tolerate a large number of needles or EA, and the utilized protocol might have been too aggressive for some patients and not sufficient for others. Yet, EA is frequently used in OA research because it has been shown to alleviate inflammatory and neuropathic pain [12, 14].

The frequency of treatments also varies greatly between protocols. Available veterinary studies in dogs have reported five treatment sessions of dry-needle acupuncture in 1 month for hip OA [22], a single treatment of EA for elbow OA [19], once weekly treatments until improvement and then every other week for multiple neurologic and musculoskeletal diseases [21], and once weekly for at least three treatments or until satisfactory improvement for chronic OA [18]. Again, while it is possible that a greater treatment frequency may result in better results, this approach may not be clinically feasible given the cost and time requirements for performing acupuncture.

The majority of the patients in this study had OA in multiple limbs/joints, which makes it more challenging to identify changes in gait analysis parameters. Yet, this is the population frequently treated with acupuncture. Future studies may consider investigating single joint OA; however, identification of a large enough sample size of single joint OA can be challenging.

## Conclusions

In this study model, acupuncture did not improve weight-bearing on the primarily affected limb, increase activity as measured by accelerometry, or improve subjective orthopedic scores; however, an owner-perceived improvement in pain and function was identified.

## Methods

This was a prospective, randomized, caregiver-blinded, placebo-controlled, cross-over design clinical trial. An a priori protocol was prepared and tested on pilot dogs prior to enrollment. This resulted in minor changes to the protocol. All enrolled cases followed the study protocol as described below.

Client-owned dogs with OA of the carpus, elbow, shoulder, tarsus, stifle, or hip joint of any breed or sex presenting to Colorado State University Veterinary Teaching Hospital were eligible for participation. The following inclusion criteria were used: body weight ≥ 10 kg, > 1 year of age, generally healthy on physical examination as well as unremarkable or stable and clinically insignificant changes on complete blood count and serum chemistry, a subjectively identifiable lameness (determined by a specialist in veterinary surgery and sports medicine; ≥2/5 but < 5/5 scores on a previously reported subjective lameness scale [28]) clearly attributable to a pelvic or thoracic limb lameness which can also be identified on OGA (values of the most affected limb had to be outside of normal range for %BWD of internal reference data), radiographically confirmed OA (greater than 6 months prior to enrollment), stable orthopedic disease (i.e. consistent lameness that has remained unchanged clinically for at least 4 weeks), a CBPI PSS and PIS ≥ 2 each [32], and willingness of the patient to tolerate the placement of one needle in GV-20 (to test for needle reactivity). If multiple limbs were affected, enrollment was based on the most severely affected limb/joint. To allow for placement of an accelerometer collar, dogs were also required to tolerate wearing a collar at all times. Dogs receiving medical management must have had a consistent OA management protocol for at least 4 weeks prior to inclusion into the study. Treatment protocols for management of OA were allowed to vary from patient to patient, but were to remain consistent throughout the study period: owners were asked to continue the current OA treatment protocol unless side effects were observed or clinical worsening required changes. Dogs were screened for study eligibility at their initial presentation, and if changes to their current treatment were suggested, all such treatments and medications were discussed with their owners. Owners were given the option to pursue the suggested changes, with re-evaluation performed a minimum of 4 weeks later to determine if the dog met the inclusion criteria.

Dogs with evidence of systemic disease (e.g. endocrinopathies), neoplasia, immune-mediated disease, lameness that was not attributable to radiographically confirmed OA, clinically significant orthopedic disease (other than OA), and evidence of neurologic disease based on a cursory neurologic examination were excluded. Animals with OA of the stifle joint due to chronic, stable cranial cruciate ligament disease were eligible; however, dogs with palpable stifle instability, lameness due to acute worsening of a suspected cranial cruciate ligament injury, or lameness due to recent meniscal injury were not eligible for enrollment. Dogs that underwent an orthopedic surgical procedure or any intra-articular injection within 6 months prior to evaluation were also ineligible for enrollment. If the owner was unable or unwilling to follow the proposed visit schedule, or fill out questionnaires, the dog was ineligible for enrollment.

### Treatment groups

**Fig. 1:**
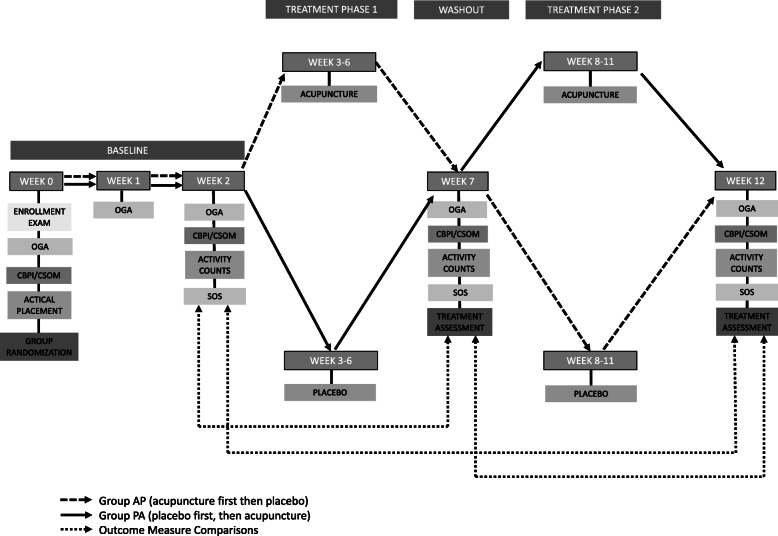
Timeline of cross-over study

Each dog was randomly allocated into one of two groups (acupuncture during the first phase and placebo during the second phase [AP], or placebo during the first phase and acupuncture during the second phase [PA]) by drawing color-coded assignments to ensure allocation of equal numbers of animals per group. This was done after enrollment to ascertain allocation concealment. Baseline data were acquired for at least 3 weeks before initiation of treatment (starting with week 0). The placebo/treatment phases consisted of weekly visits for 4 weeks. One week after completion of the placebo/treatment phase, outcome data were collected to assess treatment efficacy of each respective phase. This resulted in a minimum of 2 weeks between the final placebo/treatment and the first placebo/treatment of the next phase (see the timeline in Fig. 1). Physical and orthopedic examinations were performed at each visit prior to treatment to evaluate for any change in clinical status. Caregivers were blinded to which group their dog was assigned. Staff that received or discharged the dogs, or performed and assisted in the examinations or acupuncture treatments were not blinded; however, the acupuncturist only had contact with the caregivers during the final baseline evaluation and at the time of study completion.

#### Acupuncture protocol

Prior to study initiation, a survey was conducted to poll veterinary acupuncturists about the most effective acupuncture treatment protocol for OA-associated pain. The purpose of this survey was to aid in the development of our acupuncture protocol that would incorporate the points and procedures commonly used by veterinary acupuncturists from multiple training programs. This survey was sent to program directors of all United States veterinary acupuncture training programs to be distributed via email to their graduates. The survey was distributed to an unknown number of veterinary acupuncturists and was completed by 226 veterinarians (56% Chi Institute, 38% Curacore/Medical Acupuncture for Veterinarians, 6% International Veterinary Acupuncture Society) between 6/1/2016 and 8/24/2016. The survey consisted of 20 questions inquiring about demographics, protocols for treating arthritis, and specific acupuncture points used to treat OA patients. Questions were comprised of a combination of multiple choice and open-ended questions where additional points could be listed and commentary could be made by survey responders. For example, when practitioners were asked to list specific points for treatment of an affected joint, choices of regional points were provided by multiple choice answer in addition to a comment box where points could be manually entered. Practitioners were asked to list points by their common alpha-numeric codes for ease of analysis. Points were tallied by combining multiple choice data and word recognition counts verified by manual counts of the entries in the comment boxes. When Chinese names were provided instead of alpha-numeric codes, these were manually converted when possible to their alpha-numeric codes or tallied manually when names were unique. The treatment protocol utilized for this project (Table 2) was based on a combination of these survey results (Table 3) and protocols recommend in the published literature [18, 19, 22, 34].

**Table 2:** Acupuncture points used in the present study based on the affected joint(s)

Affected Joint	Acupuncture points in all dogs (placed bilaterally if paired)	Points for all pelvic/thoracic limb OA (placed bilaterally)	Electro-acupuncture over the primarily affected joint (unilateral EA)	Additional points used for each primarily affected joint (bilaterally placed)
Hip	GV-20^a^ BL-11—BL-23 GV-14^a^—Bai hui^a^	BL-40, ST-36, BL-54, BL-60, KID-3, LIV-3	GB-29—GB-30	Jian jiao, GB-41
Stifle			SP-10—GB-34	SP-9, GB-33, KID-3
Tarsus			BL-60—LIV-3	ST-41, ST-44, SP-6
Shoulder		BL-13, GB-21, LI-4, SI-9, BL-10	TH-14—LU-1	LI-15, SI-3, TH-3
Elbow			LI-11—LU-5	LI-10, TH-10, SI-8
Carpus			PC-6—LI-4	TH-5, LU-7, SI-3

All dogs were treated first with GV-20. The remaining points were not placed in any specific order; electro-acupuncture leads were connected between the two points (indicated by “—”) after all of the needles had been placed. Based on myofascial palpation of individual patients, 0–3 additional points not included in this table were allowed to be placed

— electro-acupuncture lead connections between these points, ^a^non-paired points

Alpha-numeric acupuncture point abbreviations [33]: *GV* Governing Vessel, *BL* Bladder, *ST* Stomach, *KID* Kidney, *LIV* Liver, *GB* Gall Bladder, *LI* Large Intestine, *SI* Small Intestine, *SP* Spleen, *TH* Triple Heater, *LU* Lung, *PC* Pericardium, # is the location on the meridian/channel

**Table 3:** Survey of veterinary acupuncturists

Abbreviated Survey Question	Survey Responses		
Which is more effective at treating OA: EA, manual acupuncture, or combination?	72% combination	21% manual acupuncture alone	7% EA alone
Do you generally use the same regional points around an affected joint, or does it vary depending on the patient?	89% variable	7% same	
With OA of a single joint, how many individualized regional points do you use on average in addition to “usual points”?	45% 1–3 additional	32% 4–6 additional	
With OA of a single joint, do you typically only acupuncture the affected limb (unilateral), or also treat the opposite limb (bilateral)?	64% treat bilaterally	15% treat only the affected limb	
With OA of a single joint, do you typically treat for compensatory pain distant from source of lameness in addition to the primary source of the lameness?	99% yes	1% no	
List the most common points you use to treat for compensatory pain based on thoracic or pelvic limb lameness.	10 most frequently mentioned for the thoracic limb: GV-14, BL-11, BL-13, GB-21, LI-4, SI-9, BL-10, LI-10, LI-11, LIV-3	10 most frequently mentioned for the pelvic limb: ST-36, BL-23, BL-40, BL-54, BL-60, GB-29, GB-30, BL-11, KID-3, and GV-14	
What regional points do you most commonly use for specific joints of the thoracic limb?	Shoulder: SI-9 (71%), GB-21 (67%), BL-11 (57%), TH-14 (63%), LI-15 (61%), and BL-10 (24%).	Elbow: LI-11 (75%), LI-10 (62%), LU-5 (43%), SI-9 (40%), TH-10 (38%)	Carpus: PC-6 (61%), HT-7 (56%), LI-4 (61%), LU-7 (38%), SI-3 (32%), and TH-5 (26%)
What regional points do you most commonly use for specific joints of the pelvic limb?	Hip: GB-29 (93%), GB-30 (92%), BL-54 (89%), and Bai hui (81%)	Stifle: ST-36 (92%), BL-40 (73%), GB-34 (67%), SP-9 (57%), ST-35 (51%), SP-10 (46%)	Tarsus: BL-60 (77%), KID-3 (69%), LIV-3 (53%), ST-36 (50%), SP-6 (38%), and ST-41 (30%)
Ranking of most to least ‘responsive to acupuncture’ arthritic joint in the appendicular skeleton.	1. Hip 2. Stifle 3. Shoulder 4. Elbow 5. Tarsus 6. Carpus		
Which points do you recommend using for every patient with OA?	Bai hui (67%), GV-20 (38%), ST-36 (51%), BL-11 (26%), BL-23 (40%), LIV-3 (23%)	10% none	

Acupuncture treatments were performed by a single certified veterinary acupuncturist once weekly for 4 weeks during the treatment phase of the study using commercially available acupuncture needles (Hwato needles, Suzhou, China; uncoated, size 0.25x30mm, 0.22x30mm, and 0.22 × 15 mm). The number of needles placed per treatment varied between 20 and 30 needles per animal depending on the protocol for the affected joint (Table 2). Needles were stimulated by rotating in a clockwise and counter-clockwise motion as the needle was advanced to attempt to achieve needle grasp (also known as “De Qi” in Chinese medicine). Duration of needle placement was between 15 and 20 min. Leads from an electro-stimulator (JM-2A Electro-acupuncture, Wuxi Jiajian Medical Instrument, Inc., Wuxi, China) were connected (according to Table 2) after all needles were placed. The points were stimulated continuously at 2 Hz for 5–10 min, followed by alternating wave pulses at 80 and 120 Hz for 5–10 min. An attempt was made to replace needles if they fell out prior to the first 10 min after needle placement. Animals were monitored for adverse effects and the treatment was terminated if the patient was showing any signs of discomfort as judged by the acupuncturist based on clinical experience.

#### Placebo protocol

The duration of the placebo treatment visits (time away from the owner) was the same amount of time as the acupuncture treatment visits. No sham acupuncture (i.e. insertion of needles into non-acupuncture points) treatment was performed; however, the patient was kept in the same room where the acupuncture was performed, unkenneled and with a staff member that also assisted in the acupuncture treatments, for the same period of time it would take to perform the acupuncture. The acupuncturist did not have contact with the patients during the placebo phase of the study and to ascertain owner blinding, patients were always received by a staff member other than the acupuncturist.

### Outcome measurements

#### Objective gait analysis

Objective gait analysis using a pressure sensitive walkway (Tekscan HRV Walkway 6 VersaTek system, Tekscan Inc., South Boston, MA) analysis system were collected at week 0 for enrollment, week 1 for acclimation, week 2 for baseline, and during the final visit of each phase of the study (week 7 and week 12). Dogs were evaluated at the trot; however, if animals were unable to trot, they were evaluated at a walk or pace. The OGA data were collected using a previously described protocol [28]. The following parameters were calculated and averaged from six valid trials of each OGA measurement for the most affected limb: PVF and vertical impulse (VI) normalized by body weight (PVF%, VI%), %BWD, and asymmetry indices (ASI) as previously described [35]. These were calculated using the following formulas:

$$ \mathrm{PVF}\%=\mathrm{PVF}\ \left[\mathrm{N}\right]/\left(\mathrm{BW}\ \left[\mathrm{kg}\right]\ \mathrm{x}\ 9.8066\right)\ \mathrm{x}\ 100 $$

$$ \mathrm{VI}\%=\mathrm{VI}\ \left[\mathrm{N}\ \mathrm{x}\ \sec \right]/\left(\mathrm{BW}\ \left[\mathrm{kg}\right]\ \mathrm{x}\ 9.8066\right)\ \mathrm{x}\ 100 $$

$$ \%\mathrm{BWD}=\left(\mathrm{PVF}\ \left[\mathrm{N}\right]\ \mathrm{of}\ \mathrm{the}\ \mathrm{limb}/\mathrm{total}\ \mathrm{PVF}\ \left[\mathrm{N}\right]\ \mathrm{of}\ \mathrm{all}\ \mathrm{four}\ \mathrm{limb}\mathrm{s}\ \mathrm{in}\ \mathrm{one}\ \mathrm{gait}\ \mathrm{cycle}\right)\ \mathrm{x}\ 100\mathrm{ASI}\ \left(\%\right)=\left(\mathrm{FL}-\mathrm{HL}/\mathrm{FL}+\mathrm{HL}\right)\ \mathrm{x}\ 100 $$

#### Accelerometry

Total AC were measured via accelerometry (Actical, Murrysville, PA, USA). The accelerometer was attached to an individual collar (in addition to the dog’s regular collar) after removing the metal ring on the collar used for leash attachment and securing the accelerometer with two zip ties. Data were recorded continuously throughout the study period. The epoch, or window length of measurement by the device, was set to 60 s. Only AC data with a minimum of 140 min of recorded activity per day were used for analysis [28]. Automatically generated data from each patient’s accelerometer was recorded as the number of minutes per week spent in different activity intensities (sedentary, light, moderate, and vigorous) [28]. Vigorous activity was not included in the statistical analysis due to the majority of patients having spent zero time in this category.

#### Subjective Orthopedic Score (SOS)

A specifically developed orthopedic scoring system (Additional file 1) was used to quantify veterinary exam findings. The SOS was modified based on previous subjective scoring systems [7, 36–40]. It consists of 6 components and each component is rated on a scale of 0–4 (with 0 = normal and 4 = severe impairment) resulting in a total score of 0–24 (with 24 being the worst). The SOS was performed by the same observer for each patient at each time point (to avoid inconsistencies in between observers assigning the scores). An attempt was made to blind the observer, however, due to the clinical proximity of this observer to the acupuncturist, this was not accomplished in several cases. As such, this study was not considered double-blinded.

#### Owner-completed questionnaires

The CBPI and CSOM questionnaires were filled out by the same owner in a dependent fashion on week 0 for enrollment, week 2 for baseline, week 7 for the end of the first placebo/treatment phase of the study, and week 12 for the end of the second placebo/treatment phase of the study (see Fig. 1). CBPI questionnaires were conducted as described previously [32]. CSOM questions based on previously published information were divided into activity and behavior categories [41]. Owners were asked to pick five time and place specific questions related to activity and grade them on a 1–5 scale (1 = no problem, 2 = a little problematic, 3 = quite problematic, 4 = severely problematic, and 5 = impossible) and three questions related to behavior also graded on a 1–5 scale (1 = significantly less than normal, 2 = less than normal, 3 = normal amount, 4 = more than normal, and 5 = significantly more than normal). These questions were normalized for analysis with higher numbers indicating worsening of symptoms and lower numbers indicating improvement of symptoms.

Additionally, owners were asked to complete a treatment efficacy form (Additional file 2) at week 7 and week 12 to record their impression of whether clinical signs and behavior changed (improved, stayed the same, worsened, or unsure of change) following each phase of the study. A final question was asked at the end of the study to assess which treatment group they felt their dog had been allocated to (assuming that the treatment would be beneficial) and whether or not the owner thought the dog improved at all during the study.

### Statistical analysis

Open-source software (http://hedwig.mgh.harvard.edu/sample_size/js/js_crossover_quant.html) was used to calculate the sample size using baseline data from two pilot dogs that were not included in the data analysis. To detect a 5% change in %BWD (significance level: 0.05; within patient standard deviation: 1.14; power: 0.9; minimal detectable difference in means: 1.25), 20 patients would have to be enrolled. To account for attrition and since this cross-over design has not been extensively used in canine OA research, we enrolled 36 dogs. Analysis was performed using SAS v9.4 (SAS Institute Inc., Cary, NC). Residual diagnostic plots were used to evaluate model assumptions (normality and equal variance). Objective gait analysis (%BWD, PVF%, VI%, and ASI [PVF contralateral, PVF ipsilateral, PVF ipsilateral, VI contralateral, VI ipsilateral, VI diagonal]), AC (Total AC, sedentary, light, and moderate), SOS, and CMI (CBPI, and CSOM) were analyzed using a mixed model that was fit separately for each response variable at baseline, post-acupuncture, and post-placebo (treating visit as a fixed effect). Each individual dog was included as a random effect to account for repeated measures. Tukey adjusted pairwise comparisons were used to compare between visits. With the exception of the primary outcome (%BWD), Bonferroni adjusted F-tests were used to account for multiple testing of the other variables. The Wilcoxon signed rank test was used to evaluate the treatment efficacy questionnaire since this data was not normally distributed. Bonferroni adjusted pairwise comparisons are also presented for the questionnaire data. A paired t-test was used to compare enrollment versus completion weights.

## Supplementary information

**Additional file 1.** Subjective Orthopedic Score (SOS).

**Additional file 2.** Owner assessment of treatment efficacy.

**Additional file 3.** Individual dog table

## Data Availability

The datasets used and analyzed during this study are available from the corresponding author on reasonable request.

## Abbreviations

%BWD: % body weight distribution
AC: Activity count
AP: Acupuncture followed by placebo
ASI: Asymmetry index
CBPI: Canine brief pain inventory
CCK: Cholecystokinin
CMI: Clinical metrology instruments
CSOM: Client specific outcome measures
EA: Electro-acupuncture
GRF: Ground reaction forces
NSAIDs: Non-steroidal anti-inflammatory drugs
OA: Osteoarthritis
OGA: Objective gait analysis
PA: Placebo followed by acupuncture
PIS: Pain interference score
PSS: Pain severity score
PVF: Peak vertical force (%: normalized to body weight)
SOS: Subjective orthopedic score
VI: Vertical impulse (%: normalized to body weight)
