# Supplementary material for: Evaluation of acupuncture for the treatment of pain associated with naturally-occurring osteoarthritis in dogs: a prospective, randomized, placebo-controlled, blinded clinical trial
Source: BMC Vet Res. 2020 Sep 25;16:357. doi: 10.1186/s12917-020-02567-1 (PMC7517673; doi:10.1186/s12917-020-02567-1)
Supplement: Supplementary file 1 — Additional file 1. Subjective Orthopedic Score (SOS). [file 12917_2020_2567_MOESM1_ESM.docx]

Additional File 1:

Subjective Orthopedic Score (SOS):

- - - **Lameness at a walk**
      1. Normal
      2. Slight lameness (inconsistent lameness/lameness is difficult to observe or it is difficult to determine the affected limb)
      3. Moderate lameness (clearly detectable lameness associated with minor head movement/pelvic tilt/forward or backward shifting of weight distribution [for bilateral lameness])
      4. Severe lameness (clearly detectable lameness associated with obvious head movement/pelvic tilt/forward or backward shifting of weight distribution [for bilateral lameness])
      5. Non-weight bearing lameness (regularly skips a few steps)
    - **Lameness at a trot**

1. Normal
2. Slight lameness (inconsistent lameness/lameness is difficult to observe or determine the affected limb)
3. Moderate lameness (clearly detectable lameness associated with minor head movement/pelvic tilt/forward or backward shifting of weight distribution [for bilateral lameness])
4. Severe lameness (clearly detectable lameness associated with obvious head movement/pelvic tilt/forward or backward shifting of weight distribution [for bilateral lameness])
5. Non-weight bearing lameness (regularly skips a few steps)
   - - **Weight bearing on the (most severely) affected limb while standing**

Score 0 = Normal

Score 1 = Slight shifting of weight bearing (inconsistent

favoring/uncertain)

Score 2 = Moderate shifting of weight bearing (unweighs affected limb[s]

but places significant weight on limb)

Score 3 = Severe shifting of weight bearing (toe-touching on affected

limb[s] but remains in contact with floor)

Score 4 = Non-weight bearing on affected limb[s] for more than 50% of

the observed time while standing

- - - **Pain on manipulation** (during ROM of the affected joint – *Note: select the ‘worse category’ based on the assessments made; i.e. if only one of two criteria are present select the worse category: example - if a dog has normal ROM but mild pain response – select ‘mild pain’)*

Score 0 = Normal (=full ROM; no pain on ROM; does not notice or resist

manipulation in any form)

Score 1 = Mild pain (=slightly reduced ROM; no definitive or mild pain

response, i.e. may orient head towards side of manipulation,

but does not resist joint manipulation)

Score 2 = Moderate pain (=slight to moderate reduction in ROM;

moderate pain response such as withdrawing limb, moving away, licking and slight objection to manipulation)

Score 3 = Severe pain (=slight to severe reduction in ROM; severe pain

response such as vocalizing, biting, sudden turning of head

towards manipulation, withdrawing from manipulation and

excessive licking OR moderate pain response during minimal

ROM)

Score 4 = Excessive pain (=severe reduction in ROM; excessive pain

indicated by symptoms such as will not let observer manipulate

joint, tries to escape from manipulation, or prevent

manipulation, may bite or show aggression on manipulation)

- - - **Willingness to lift the contralateral limb (veterinarian lifts up contralateral limb)**

Score 0 = Normal – readily lifts up contralateral limb and does maintain

standing on affected limb for >15 seconds

Score 1 = Readily lifts up contralateral limb but attempts to replace limb

after 10 seconds

Score 2 = Readily lifts up contralateral limb but attempts to replace limb

immediately

Score 3 = Offers clear resistance to lifting up contralateral limb

Score 4 = Refuses to lift contralateral limb

- - - **Functional disability (independent of lameness)**

*NOTE: This scoring is designed to identify disability related to multiple affected limbs. For example, a healthy dog with an isolated problem (such as unilateral CCLD) would score 0, a dog with bilateral, symmetric HD may score 1-4 but not actually have a unilateral lameness*

Score 0 = Normal gait of unaffected limbs; no stiffness

Score 1 = Mild functional disability; slightly stiff gait noticeable when

trotting but not at a walk

Score 2 = Moderate functional disability; dog has noticeable stiffness

while walking and trotting

Score 3 = Severe functional disability; dog walks but does not want to trot

without being coaxed

Score 4 = Complete disability; dog does not want to walk or trot
