# Supplementary material for: Evaluation of acupuncture for the treatment of pain associated with naturally-occurring osteoarthritis in dogs: a prospective, randomized, placebo-controlled, blinded clinical trial
Source: BMC Vet Res. 2020 Sep 25;16:357. doi: 10.1186/s12917-020-02567-1 (PMC7517673; doi:10.1186/s12917-020-02567-1)
Supplement: Supplementary file 2 — Additional file 2. Owner assessment of treatment efficacy. [file 12917_2020_2567_MOESM2_ESM.docx]

Additional File 2:

Owner assessment of treatment efficacy:

As you know, your dog is enrolled in a randomized, blinded clinical trial. This means that you don’t know whether your dog received treatment or placebo over the last few weeks. Now that your dog has completed the first phase of the study, we would like to seek your input on whether you believe that the treatment employed over the last few weeks helped your dog. For this purpose, please answer the two questions below:

# 1^st^ Treatment Period

1. Did you notice a change in any of the clinical signs that you believe are attributed to your dog’s osteoarthritis during the last treatment period?

- YES, my dog’s clinical signs improved
- YES, my dog’s clinical signs worsened
- NO, my dog’s clinical signs remained the same
- UNSURE -– please explain below

………………………………………………………………………………………………………………………………………………………………………………………………………………………………………………………………………………………………………………………………………………………………………………………………………………………………………………………………………………………………………………………………………………………………………………………………………………………………………………………………………………………………………………………………………………………………………………………………………………………………………………………………

1. Did you notice any other differences in your dog’s behavior that you believe could be due to the treatment administered?

- YES – I noticed a positive change (please explain below)
- YES – I noticed a negative change (please explain below)
- NO – I noticed no change
- UNSURE

………………………………………………………………………………………………………………………………………………………………………………………………………………………………………………………………………………………………………………………………………………………………………………………………………………………………………………………………………………………………………………………………………………………………………………………………………………………………………………………………………………………………………………………………………………………………………………………………………………………………………………………………………………………………………………………………………………………………………

# 2^nd^ Treatment Period

Now that your dog has completed the 2^nd^ phase of the study, we would like to seek your input on whether you believe that the treatment employed over the last few weeks helped your dog. You are welcome to review your answers above (but you may not change them). Please answer the three questions below:

1. Did you notice a change in any of the clinical signs that you believe are attributed to your dog’s osteoarthritis during the last treatment period?

- YES, my dog’s clinical signs improved
- YES, my dog’s clinical signs worsened
- NO, my dog’s clinical signs remained the same
- UNSURE -– please explain below

………………………………………………………………………………………………………………………………………………………………………………………………………………………………………………………………………………………………………………………………………………………………………………………………………………………………………………………………………………………………………………………………………………………………………………………………………………………………………………

1. Did you notice any other differences in your dog’s behavior that you believe could be due to the treatment administered?

- YES – I noticed a positive change (please explain below)
- YES – I noticed a negative change (please explain below)
- NO – I noticed no change
- UNSURE

………………………………………………………………………………………………………………………………………………………………………………………………………………………………………………………………………………………………………………………………………………………………………………………………………………………………………………………………………………………………………………………………………

1. Assuming that the treatment tested in this study is effective at reducing symptoms associated with osteoarthritis – would you be able to guess which treatment period was the placebo treatment vs. the actual treatment?

- YES (please select from one of the options below):
  - I believe my dog received treatment during the 1^st^ period and placebo during the 2^nd^ period
  - I believe my dog received placebo during the 1^st^ period and treatment during the 2^nd^ period
- NO – I don’t believe there was a difference between the two treatment periods
- UNSURE
